# Assessment of Comorbidities in Chronic Obstructive Pulmonary Disease Patients by Chest High Resolution Computed Tomography (HRCT)

**DOI:** 10.3390/jcm15020785

**Published:** 2026-01-19

**Authors:** Ecaterina Iavrumov, Dumitru Cravcenco, Alexandr Ceasovschih, Pradeesh Sivapalan, Nikos Siafakas, Alexandru Corlateanu

**Affiliations:** 1 Department of Respiratory Medicine and Allergology, State University of Medicine and Pharmacy “Nicolae Testemitanu”, 2004 Chisinau, Moldova; ecaterina.iavrumov@usmf.md; 2 Medical Center Scanexpert, 2001 Chisinau, Moldova; krav84@mail.ru; 3 Grigore T. Popa University of Medicine and Pharmacy, 700115 Iasi, Romania; alexandr.ceasovschih@umfiasi.ro; 4 Department of Clinical Medicine, Faculty of Health and Medical Sciences, University of Copenhagen, DK-2200 Copenhagen, Denmark; pradeesh.sivapalan.02@regionh.dk; 5 Copenhagen Respiratory Research, Department of Medicine, Copenhagen University Hospital—Herlev and Gentofte, DK-2900 Copenhagen, Denmark; 6 Department of Computer Science, University of Crete, 70013 Heraklion, Greece; siafakan@uoc.gr

**Keywords:** COPD, comorbidities, chest HRCT, chest computed tomography

## Abstract

**Purpose:** Chronic Obstructive Pulmonary Disease (COPD) is a progressive respiratory condition often accompanied by various comorbidities that significantly affect patient outcomes. High resolution computed tomography (HRCT) has emerged as a valuable tool for diagnosing and managing COPD-related comorbidities. This study aimed to explore the impact of chest computed tomography (CT) imaging in identifying and characterizing comorbidities in COPD patients. **Methods:** The study was conducted on 99 patients with COPD, the median age of the study population was 70.0 years (Q1–Q3: 62.0–75.0); 86% were men (85), and 14% were women (14). All patients underwent chest HRCT to identify the presence of comorbidities. **Results:** According to the GOLD classification (ABE groups), 3% were type A, 27% were type B, and 69% were type E. The prevalence of comorbidities identified on chest HRCT was reported as 66% for coronary artery calcification (CAC), 83% for osteoporosis, 36% for pulmonary artery enlargement (PAE), 31% for emphysema, 19% for bronchiectasis, 17% for hiatal hernia, 14% for lung cancer, 12% pulmonary infections, and 3% for interstitial abnormalities. In 4%, there were no comorbidities, one comorbidity was found in 11%, two comorbidities in 17%, and three comorbidities and more in 68% of cases. **Conclusions:** Chest HRCT imaging serves as a valuable tool for identifying and assessing comorbidities in patients with COPD. Incorporating chest CT imaging into the routine evaluation of COPD patients may contribute to a more comprehensive understanding of their condition and lead to better clinical outcomes.

## 1. Introduction

**Figure 1:**
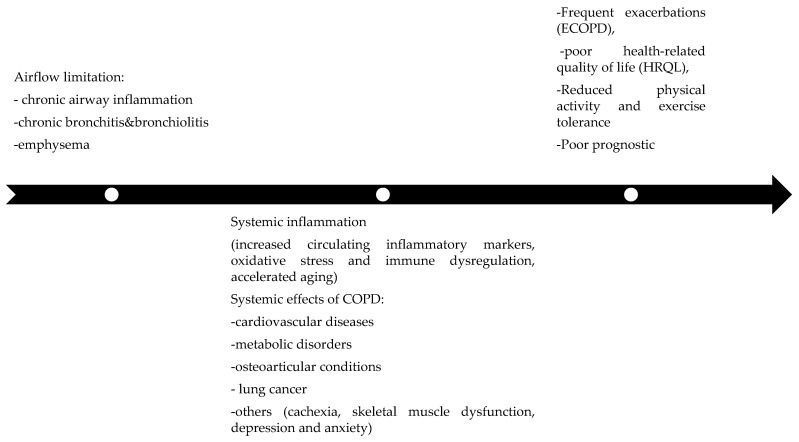
Pathophysiological continuum and clinical consequences of COPD progression. ECOPD, exacerbation of COPD; HRQL, health-related quality of life.

Chronic Obstructive Pulmonary Disease (COPD) is now one of the top three causes of death worldwide [1]. COPD represents an important public health challenge that is preventable and treatable [2]. COPD is a leading cause of morbidity and mortality globally with a huge social and economic burden [3]. The prevalence of COPD is often directly related to the prevalence of tobacco smoking [4], and occupational, outdoor, and household air pollution are important COPD risk factors [1]. According to the Global Initiative for Chronic Obstructive Lung Disease (GOLD) report, the diagnosis of COPD is based on the demonstration of persistent airflow limitation by spirometry (post-bronchodilator FEV1/FVC < 0.70), in the appropriate clinical context [1]. COPD is a complex disease with pulmonary and extra pulmonary manifestations [5,6] due to pulmonary and systemic inflammation [7]. As shown in Figure 1, COPD progression involves a complex interplay between airflow limitation, systemic inflammation, and multi-organ comorbidities.

COPD frequently coexists with other conditions known as comorbidities [8,9,10]. Some of these are identifiable using validated chest CT techniques. These include coronary artery calcification, osteoporosis, pulmonary artery enlargement, bronchiectasis, hiatal hernia, lung cancer, pulmonary infections, and interstitial abnormalities [11]. However, GOLD also emphasizes that imaging, particularly chest CT, provides complementary and clinical relevant information by enabling the assessment of structural lung abnormalities, phenotyping of COPD, and detection of associated comorbidities [1,12,13]. Annual low-dose CT scan is also recommended for lung cancer screening in heavy smokers with COPD. CT contributes to the evaluation of emphysema, airway disease, pulmonary vascular changes, and extrapulmonary manifestations, supporting a more comprehensive and personalized approach to COPD management [1].

The identification of comorbidities in COPD is a major clinical importance, as they substantially influence symptoms, exacerbation risk, response to therapy, quality of life, and survival. CT clearly has the potential to become a powerful tool in the quest of personalized medicine in COPD. This study was carried out in a cohort of COPD patients attending pulmonary clinics with the aim to assess the prevalence of comorbidities identified through CT imaging.

## 2. Materials and Methods

### 2.1. Participants

This was a retrospective, observational study involving COPD inpatients who were prospectively enrolled between 2022 and 2023. All participants had mild to severe airway obstruction. COPD was diagnosed based on the GOLD 2024 criteria. Spirometric lung function data (EasyOne Connect V3.9.4.9, ndd Medizintechnik, Zurich, Switzerland) included forced expiratory volume in 1 s (FEV1) and forced vital capacity (FVC). GOLD grades 1–4 were defined as follows: post-bronchodilator FEV1/FVC < 0.7, with GOLD grade 1 (mild) defined as FEV1 ≥ 80% predicted, GOLD grade 2 (moderate) as 50% ≤ FEV1 < 80% predicted, GOLD grade 3 (severe) as 30% ≤ FEV1 < 50% predicted, and GOLD grade 4 (very severe) as FEV1 < 30% predicted. GOLD groups A, B, and E, were based on the modified Medical Research Council (mMRC) dyspnea scale, in combination with the exacerbation risk, which was determined based on a 12-month history of exacerbations of all severities, including hospitalizations. Smoking status was classified as active smokers compared to non-smokers or ex-smokers. Exclusion criteria included uncontrolled comorbidities, such as pulmonary tuberculosis. At baseline, the following data were collected: age, gender, smoking status, and the occurrence of exacerbations in the previous year. The presence of comorbidities was recorded based on patients’ reports of physician-diagnosed conditions and information available in the medical records. The present analysis focused on the description of comorbidities identified by chest HRCT.

The research protocol was reviewed and approved by the Research Ethics Committee of the Nicolae Testemitanu State University of Medicine and Pharmacy, Chisinau, Republic of Moldova on 31 March 2022 (Approval No. 30), in accordance with the ethical standards of the institution and international research guidelines.

### 2.2. Chest CT Protocol and CT-Assessed Comorbidities

The study subjects underwent imaging with 128-detector row CT scanners (SOMATOM Perspective; Siemens, Erlangen, Germany). Images were obtained during breath hold at full inspiration in the supine position. Acquisition parameters were as follows: tube voltage, 120–140 kVp; tube current, 30–100 mAs; and collimation, 0.6–1 mm. Reconstruction parameters were as follows: kernel, medium/high resolution (B50f); slice thickness, 0.75 mm; and slice interval, 1 mm. All examinations were performed using standard chest CT acquisition protocols. Low-dose screening CT was not systematically applied in this cohort. The images were reviewed by using a post processing workstation, Phillips, IntelliSpace Portal, with window level −600 HU and window with 1600 HU.

The L1 vertebra is most commonly measured because it is generally included in CT examinations of the thoracolumbar spine, chest, and abdomen. Osteoporosis is typically indicated by an attenuation of 90 HU or less (specificity > 90%). Conversely, an attenuation greater than 160 HU essentially rules out osteoporosis, with a negative predictive value of 95% [14]. For coronary artery calcification, the most commonly used method is the Agatston score, which employs a threshold of >130 Hounsfield units and an area of three or more pixels to identify calcified plaque within the track of the epicardial vessels on non-contrast CT. The calcium score can be categorized as minimal (0–10), mild (11–100), moderate (101–400), or severe (>400). PAE was defined as a PAD ≥ 29 mm in men and ≥27 mm in women.

Over the last two decades, chest CT has been the gold standard for the in vivo diagnosis of bronchiectasis. Bronchiectasis is diagnosed when an abnormally wide and often thickened airway is observed, which typically also shows a lack of tapering. Based on appearance and severity, bronchiectasis can be classified as cylindrical, varicose, or cystic.

A hiatal hernia (HH) is the herniation of abdominal organs and structures through the esophageal hiatus of the diaphragm into the thoracic cavity. There are four types of HH: sliding (type 1), paraesophageal (rolling) (type 2), mixed (type 3), and mixed type with herniation of visceral organs (type 4).

Chest CT remains the standard imaging test for the initial assessment of suspected lung cancer. The use of thin-section multi-detector CT often detects numerous pulmonary nodules. Nodule characterization on CT relies on morphological criteria, such as irregular or spiculated margins for malignancy and calcifications for benignity. Hilar tumors are those with the center of mass within or proximal to the bronchial lumen, while peripheral tumors are distal to a segmental bronchus. Lymph nodes are considered enlarged if their short-axis diameter is ≥10 mm.

The typical appearance of acute community-acquired pneumonia (CAP) is a single subpleural area of alveolar consolidation with blurred margins, restricted to the region next to the fissures. It progresses to a systematized segmental opacity affecting one or several contiguous segments or a lobe, with or without the air bronchogram sign. Ground-glass opacities may also be present adjacent to the alveolar consolidation due to partial alveolar filling [15].

ILA is defined as incidental CT findings of nondependent abnormalities affecting more than 5% of any lung zone (upper, middle, and lower lung zones are demarcated by the levels of the inferior aortic arch and right inferior pulmonary vein). These findings include ground-glass or reticular abnormalities, lung distortion, traction bronchiectasis or bronchiolectasis, honeycombing, and nonemphysematous cysts.

**Figure 2:**
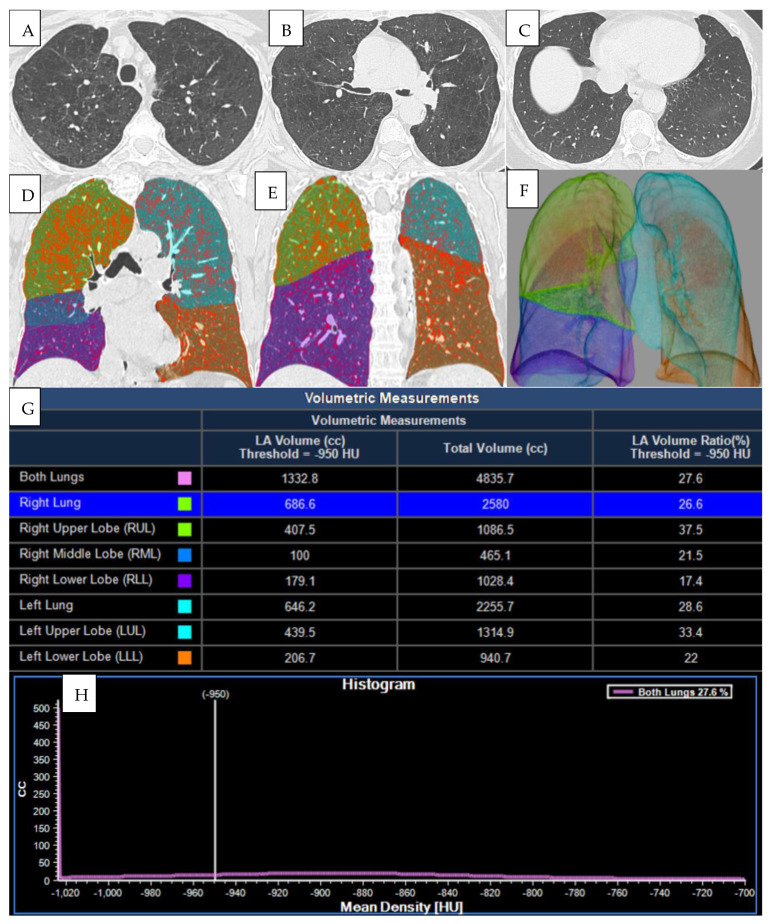
Quantitative and volumetric HRCT assessment of emphysema. (**A**–**C**) Axial CT scans demonstrating paraseptal and centrilobular emphysema. (**D**–**F**) Three-dimensional segmentation and lobar volumetry for emphysema quantification. Computed tomography (CT) provides a detailed evaluation of emphysema distribution and burden in patient with COPD. (**G**,**H**) Voxels with attenuation values below a predefined threshold (typically set at −950 HU) are classified as emphysematous. This threshold facilitates the segmentation of lung tissue into emphysematous and non-emphysematous regions, thereby enabling a quantitative assessment of the proportion of lung volume affected by emphysema.

Chest computed tomography (CT) is a non-invasive imaging modality that offers valuable insight into structural and pathophysiological pulmonary parameters, enhancing the understanding of disease variability and further characterizing COPD phenotypes, such as emphysema, airway disease, and/or air trapping [16]. Quantitative CT evaluation can effectively identify emphysema. The visual classification system from the Fleischner Society grades the severity of parenchymal (nonparaseptal) emphysema as trace, mild, moderate, confluent, and advanced destructive emphysema, as well as paraseptal and panlobular emphysema. Quantitative CT evaluation can affectively identify emphysema and visualize different morphological patterns, as shown in Figure 2.

### 2.3. Statistical Analysis

Categorical data are presented as frequency, percentage (%) and were compared using the Chi square test or Fisher’s exact test, as appropriate. Means were compared using the independent sample *t*-test and medians using the Mann–Whitney U test. Continuous variables were tested for normality using Shapiro–Wilk test. Normally distributed data are presented as mean ± standard deviation, while non-normally distributed data are expressed as median (Q1–Q3). Other predictors were included according to the specific study question (see Section 3). All analyses were performed with the software SPSS Statistics V22.0 (IBM Corp., Armonk, NY, USA).

## 3. Results

The study included 99 patients with COPD. The median age of the study population was 70.0 years (Q1–Q3: 62.0–75.0). Of the participants, 86% were men and 14% were women. Baseline demographic and functional characteristics of the study cohort are summarized in Table 1.

The prevalence of HRCT-detected comorbidities among COPD patients is summarized in Table 2.

**Figure 3:**
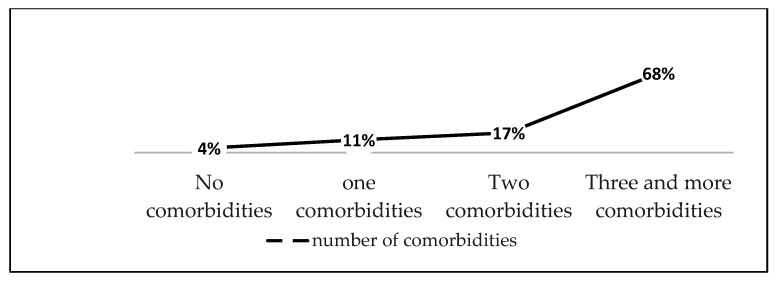
Frequency of comorbidities.

According to Figure 3, the majority of patients exhibited multimorbidity, emphasizing the systemic nature of COPD.

**Figure 4:**
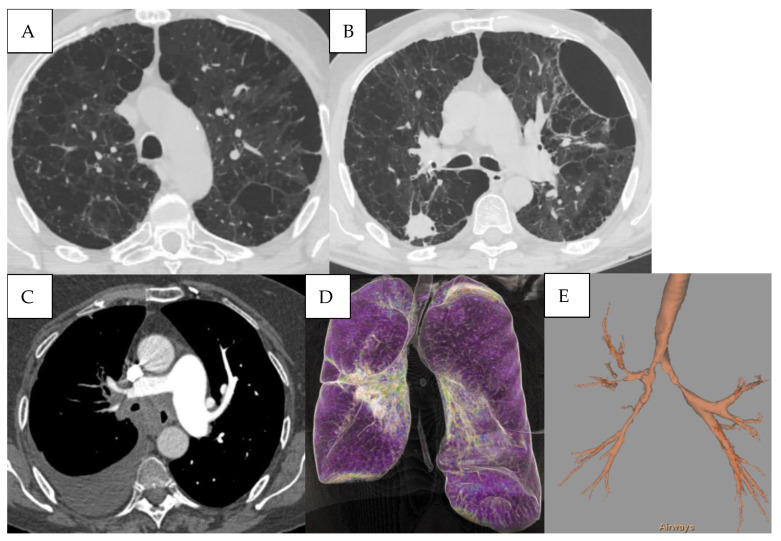
(**A**) Mixed bilateral pulmonary emphysema (paraseptal and centrilobular emphysematous bullae). (**B**) Peripheral nodular formation in the lower lobe of the right lung. Mixed bilateral pulmonary emphysema. (**C**) Central tumoral mass of the right lung with invasion of the main bronchi bilaterally and lobar branches B4, B5 on the right. (**D**,**E**) Virtual bronchoscopy, presence of extrinsic stenosis of the main bronchi bilaterally.

Detailed HRCT examples of emphysema morphology and bronchial stenosis are presented in Figure 4.

**Figure 5:**
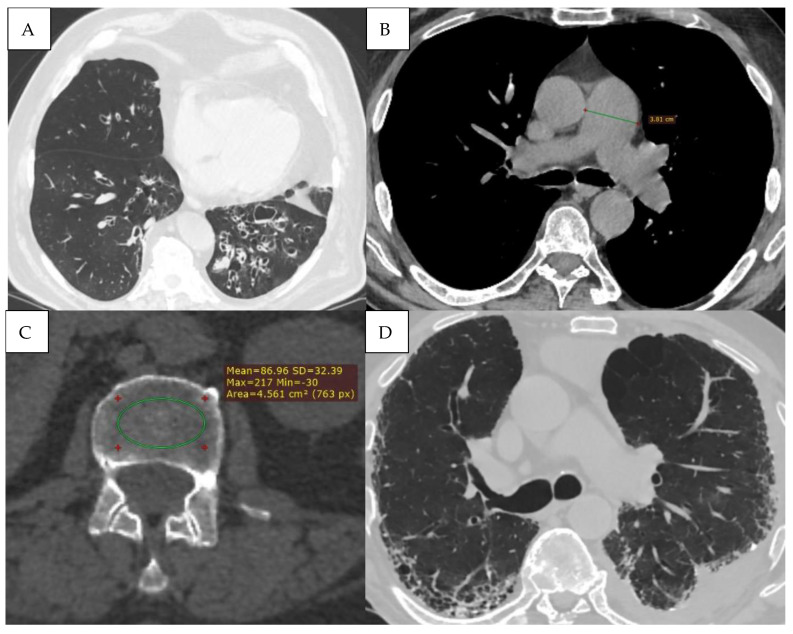
(**A**) Bronchiectasis (multiple types of bronchiectasis in the lower lobe of the right lung, some with mucoid impaction). (**B**) CT signs of pulmonary hypertension (diameter of the pulmonary trunk was 3.8 cm (normal < 2.9 cm)). (**C**) Osteoporosis, bone density measurement at the level of L3 (86.96 HU). (**D**) ILD (Interstitial Lung Disease), subpleural honeycombing formed by clustered cysts stacked together in several layers. Reticular pattern. Left pleural effusion.

As depicted in Figure 5, chest CT further revealed a wide spectrum of comorbidities, including bronchiectasis, pulmonary artery enlargement, osteoporosis, and interstitial lung disease.

**Figure 6:**
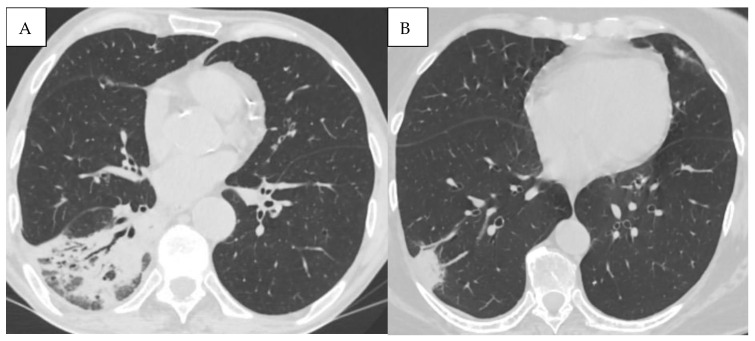
(**A**) Pneumonia—Pulmonary consolidation in the lower lobe of the right lung, with the presence of air bronchogram. (**B**) Pulmonary infarction represented by the peripheral wedge-shaped pulmonary consolidation in the right lung with its base towards the pleura.

Further CT examples of infectious and vascular comorbidities are presented in Figure 6.

**Figure 7:**
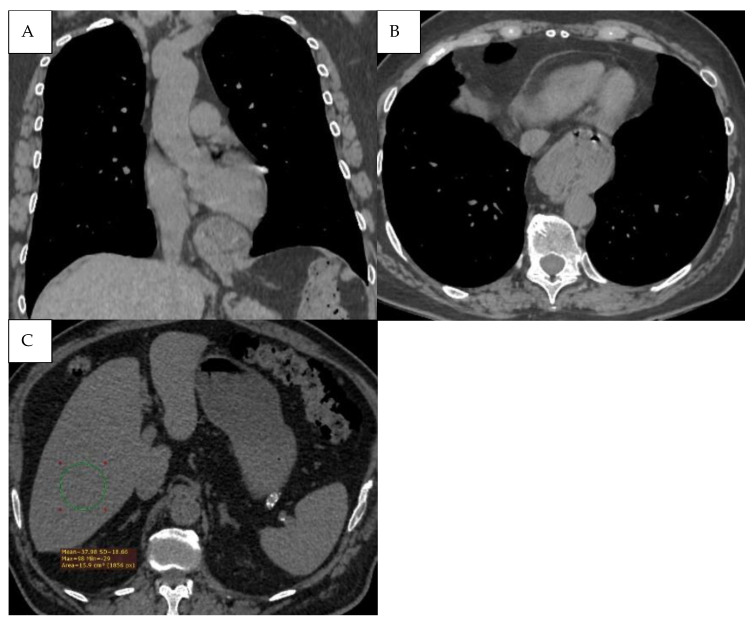
(**A**,**B**) CT images shows a hiatal hernia (type 1), with a part of the stomach protruding through the esophageal hiatus into the thoracic cavity. (**C**) Abdominal CT image showing hepatic steatosis with diffusely decreased liver attenuation (37.98 HU). The green circle indicated the region of interest for liver attenuation measurement.

As demonstrated in Figure 7, HRCT also revealed extrapulmonary changes, including hiatal hernia and hepatic steatosis, further supporting the systemic nature of COPD.

**Figure 8:**
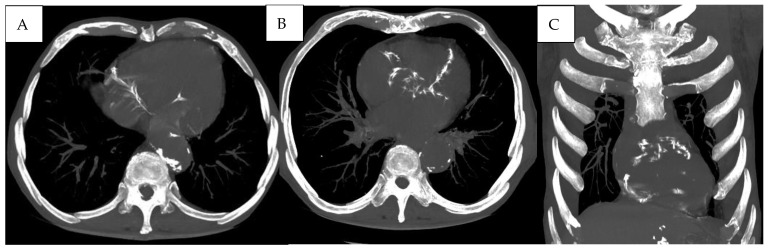
(**A**,**B**) Multiple vascular calcifications of the thoracic aorta and proximal coronary arteries are visible, suggesting underlying atherosclerotic cardiovascular disease. (**C**) Coronal reconstruction further highlighting the presence of extensive aortic and coronary calcifications.

Advanced pulmonary vascular changes and cardiovascular calcifications were observed on CT, as illustrated in Figure 8.

## 4. Discussion

In this study, we observed that patients with COPD, based on the analyzed variables and the data obtained from computed tomography, showed a higher prevalence of imaging-based comorbidities compared to the comorbidities established through clinical criteria.

Osteoporosis is a significant comorbidity in COPD patients. The World Health Organization (WHO) defined osteoporosis as ‘‘a disease characterized by low bone mass and micro-architectural deterioration of bone tissue, leading to enhanced bone fragility and a consequent increase in fracture risk’’ [17]. We found that 83% of the patients with COPD had CT-defined osteoporosis, a higher prevalence similar to other studies [18,19,20,21]. Osteoporosis affects a significant proportion of patients with COPD. Studies have reported prevalence rates ranging from 35% to over 70%, depending on disease severity, population characteristics, and diagnostic modalities used [22,23]. In the ECLIPSE study, which included a large international COPD cohort, osteoporosis was present in approximately 69% of participants when evaluated with DXA (dual-energy X-ray absorptiometry) and CT indicators [23]. The identification of osteoporosis via chest CT), routinely performed in COPD management, has garnered attention for its ability to uncover subclinical bone loss not otherwise captured by traditional methods such as DXA. Graat-Verboom et al. demonstrated that thoracic CT could predict osteoporosis in 80% of COPD outpatients, supporting its role in comorbidity screening [18]. The gold standard for diagnosing osteoporosis is DXA and DXA of the lumbar spine and hip being the most common method [24].

COPD and coronary artery calcification (CAC) were found in 66% of cases. Multiple studies have demonstrated a higher prevalence of CAC in COPD patients compared to age- and smoking-matched controls. In the ECLIPSE study, which included over 2000 subjects, CAC was observed in up to 67% of COPD patients, significantly higher than in matched controls without airflow limitation [25]. Similarly, in the MESA-Lung Study, the prevalence of CAC (Agatston score > 0) among COPD patients reached 70%, and the severity of calcification was associated with worse lung function [26]. Coronary artery calcification can be identified by CT and is pathognomonic of coronary atherosclerosis. When considering CT for COPD phenotyping, coronary vessel wall calcification could be a potential marker of cardiac disease [27]. Both diseases have a high worldwide prevalence and frequently coexist [27]. Coronary vessel wall calcifications are an independent risk factor for mortality in patients with and without COPD. Therefore, it is considered to be clinically valuable to quantify this finding and estimate the individual risk for cardiac ischemia in COPD patients presenting with calcification on CT [28]. A population-based study by Chiles et al. showed that CAC detected incidentally on lung cancer screening CT scans was independently associated with cardiovascular mortality, emphasizing the prognostic value of this finding in smokers, many of whom also have COPD [29].

Pulmonary artery enlargement (PAE) was established in 36%. The incidence of pulmonary hypertension (PH) varies in patients with COPD. It is usually associated with a severe degree of airway obstruction and is prevalent in 25–35% of the severely affected COPD population, but it has also been described as frequent in 5–7% of patients with only mild to moderate degree. A landmark study by Wells et al. reported a prevalence of PAE (defined as a pulmonary artery to ascending aorta diameter ratio [PA/Ao] > 1) in 50% of patients hospitalized with COPD exacerbations and in 20% of stable COPD patients [30]. Other studies reported prevalence rates ranging between 25–45%, depending on disease severity and imaging modality used [31]. In the ECLIPSE study, PAE was also found to correlate with increasing GOLD stages and was associated with a higher frequency of exacerbations and hospitalizations [32]. Right-sided heart catheterization (RHC) is considered the gold standard for diagnosing pulmonary hypertension (PH), but it is rarely performed in patients with COPD due to its invasive nature and the lack of effective treatment options. Instead, echocardiography, which estimates pulmonary artery systolic pressure (PASP), is the most commonly used noninvasive screening method for PH. However, this technique is not sufficiently accurate, particularly in COPD patients, as factors such as lung hyperinflation and adipose tissue can interfere with the quality of the echocardiographic examination [33,34].

COPD and bronchiectasis were identified in 19% cases. Both are very prevalent in the general population, and they may both coexist. Several studies have documented the presence of bronchiectasis in 15–45% of patients with COPD, with the highest prevalence observed in individuals with frequent exacerbations and moderate-to-severe airflow limitation [35]. A multicenter study by Martínez-García et al. identified bronchiectasis in 29% of COPD patients undergoing HRCT, and its presence was independently associated with a history of more than two exacerbations per year [36]. High-resolution computed tomography (HRCT) is essential in diagnosing bronchiectasis, which often remains under-recognized in patients with chronic respiratory symptoms. There is a strong possibility of misdiagnosis in favor of COPD, because spirometry is more widely available than CT scans, and physicians usually think primarily about COPD when confronted with a smoker with cough, sputum production, and airflow obstruction. Furthermore, in COPD patients with pulmonary hypertension, the diagnostic criterion for bronchiectasis (based on the demonstration of a bronchial lumen diameter greater than the diameter of the adjacent vessel) may be misleading [37].

A total of 17% of COPD patients were identified with hiatal hernia (HH). Several observational studies have reported a higher prevalence of HH in COPD patients than in the general population. In one study using chest CT in stable COPD patients, the prevalence of radiologically detected HH was approximately 17–21%, depending on the severity of disease and criteria used [38,39]. HHs are easily identified on non-contrast CT of the chest as a proximal displacement of the esophagogastric junction through the esophageal hiatus of the diaphragm into the mediastinum [40]. Since COPD patients routinely undergo CT scans of the thorax, our aim was to (1) formally define hiatal hernia seen on CT and (2) determine whether these findings can be used as a visual marker to identify patients who may be frequent exacerbators, especially given the relationship between GERD and increased COPD exacerbations [41].

Lung cancer is the second most common malignancy worldwide, resulting in one-quarter of all cancer-related deaths. Patients with COPD have a 2- to 6-fold increased risk of developing lung cancer compared to smokers without airflow limitation [42]. In this study, lung cancer (pulmonary nodules or pulmonary masses) was found in 14% of cases. In a prospective cohort study involving over 2000 participants with COPD, the 10-year cumulative incidence of lung cancer was approximately 16–20%, particularly in those with emphysema-dominant phenotypes and GOLD stage III/IV airflow limitation [43]. CT with virtual bronchoscopy (VB) has been found to be very helpful in defining the location, extent, and nature of these lesions, and is increasingly being used even in patients with contraindications for fiberoptic bronchoscopy and laryngoscopy. VB is one of the most recently developed interactive 3D techniques applicable to the tracheobronchial tree [44]. The presence of emphysema, interstitial abnormalities, or both are significant risk factors for future lung cancer development in COPD patients [45].

Infections are among the most frequent and impactful comorbidities in chronic obstructive pulmonary disease (COPD), contributing significantly to disease progression, acute exacerbations, and mortality. Both acute and chronic infections shape the clinical course of COPD, and modern imaging, especially computed tomography (CT), plays a central role in detecting infection-related structural lung changes. Respiratory infections are the leading cause of exacerbations in COPD, accounting for 50–80% of acute events. Infection as an associated condition of COPD was observed in 12% in this study. Bacterial infections are implicated in approximately 55% of exacerbations, while viral infections are found in 30–40%, often coexisting with bacterial pathogens [46,47,48]. In large COPD cohorts (e.g., ECLIPSE, SPIROMICS), annual exacerbation rates range from 0.85 to 1.3 per patient, with higher rates in patients with prior exacerbations or advanced disease [49]. Since these infections significantly affect the clinical progression of a patient with COPD, they represent a notable comorbidity in COPD. Infection and COPD seem to have a reciprocal and causative relationship, thus recognizing infection as an associated condition in COPD [50].

Interstitial lung abnormalities (ILAs) refer to specific, subtle patterns of increased lung density on chest computed tomography (CT) that are not diagnostic of interstitial lung disease (ILD) but may reflect early fibrotic changes. In patients with chronic obstructive pulmonary disease (COPD), the coexistence of ILA may define a distinct clinical and radiological phenotype with unique prognostic implications. Interstitial abnormalities in COPD were identified only in 3% of cases. The reported prevalence of ILA in COPD patients varies between 2% and 10%, depending on age, smoking status, and imaging resolution. In the COPDGene study, ILAs were identified in approximately 8% of COPD patients, while a lower prevalence (~3%) was observed in general imaging-based screening studies [51].

From a clinical perspective, the findings of this study support the value of opportunistic chest CT assessment in patients with COPD. Chest CT, frequently performed for lung evaluation or cancer screening, offers a unique opportunity to simultaneously identify cardiovascular, skeletal, pulmonary vascular, infectious, and neoplastic comorbidities which substantially influence prognosis and disease control. Systematic reporting of coronary artery calcification, pulmonary artery enlargement, osteoporosis, bronchiectasis, and interstitial abnormalities may facilitate early multidisciplinary referral, individualized risk stratification, and targeted preventive interventions. Integrating CT-derived comorbidity assessment into routine COPD evaluation could therefore contribute to more comprehensive disease management, improved phenotyping, and optimization of long-term outcomes.

### Study Limitations

The present study was designed as a descriptive imaging-based assessment of comorbidities in COPD and did not include longitudinal follow-up or outcome-based correlation. The absence of a non-COPD control group preclude causal interferences regarding the prevalence of CT-detected comorbidities. Future studies on larger cohorts are warranted to explore the relationship between COPD severity and CT-detected comorbidity burden.

## 5. Conclusions

Comorbidities are common in COPD patients, and their assessment represents an important component of comprehensive disease evaluation. The identification of comorbidities through chest CT in patients with COPD holds significant clinical value. Numerous studies have demonstrated that comorbidities such as cardiovascular diseases, lung cancer, osteoporosis, bronchiectasis, and pulmonary hypertension are prevalent in COPD patients, often contributing to worsened outcomes and increasing the burden of disease. Chest CT, with its ability to non-invasively detect a wide range of abnormalities, may represent an essential tool in the early identification and management of these comorbid conditions. For instance, the detection of coronary artery calcification and pulmonary artery enlargement via CT can provide early indications of cardiovascular disease, a common comorbidity that exacerbates the prognosis of COPD patients. Additionally, the visualization of bronchiectasis and emphysema on CT scans aids in more accurate phenotyping of COPD, allowing for personalized treatment strategies. Furthermore, CT’s role in diagnosing other significant comorbidities such as lung cancer and osteoporosis emphasizes the need for comprehensive assessment in COPD patients, as these conditions are often underdiagnosed without imaging techniques. In light of these findings, our results suggest that routine chest CT screening in COPD patients not only aids in the management of respiratory symptoms but also enhances the detection of comorbidities that may otherwise be overlooked. This integrated approach to diagnosis is crucial for optimizing patient outcomes, reducing complications, and improving the overall management of COPD. However, given the retrospective design and relatively small sample size, these findings should be interpreted with caution, and further prospective studies are warranted.

## Figures and Tables

**Table 1:** Demographic characteristics and functional data of participants at baseline (*n* = 99).

Variables		
Age in years, median (Q1–Q3)		70.0 (62.0–75.0)
Sex, *n* (%)	Male sex	86
	Female sex	14
Smoker, *n* (%)	No smoker	8.0
	Current smoker	58.5
	Ex-smoker	33.3
FEV1%, mean ± SD		57.9 ± 12.4
Spirometry GOLD stages (%)	I	9
	II	18
	III	52
	IV	21
Type group GOLD (*n* = 99), *n* (%)	A	3 (3.0)
	B	27 (27.3)
	E	69 (69.7)

Notes: Data are presented as median (Q1–Q3) or mean ± standard deviation, as appropriate. Abbreviations: GOLD, Global Initiative for Obstructive Lung Disease; FEV1, forced expiratory volume in 1 s.

**Table 2:** Prevalence of the different CT-assessed comorbidities.

Morbidity Detected	% of Patients
Osteoporosis	83
CAC	66
PAE	36
Emphysema (as a part of COPD)	31
Bronchiectasis	19
Hiatal hernia	17
Lung cancer	14
Pulmonary infections	12
ILA	3

Abbreviations: CAC, coronary artery calcification; PAE, pulmonary artery enlargement; ILA, interstitial lung abnormalities.

## Data Availability

The original contributions presented in the study are included in the article. Further inquiries can be directed to the corresponding author.

## Abbreviations

The following abbreviations are used in this manuscript:

COPD	Chronic Obstructive Pulmonary Disease
CT	Computed Tomography
DXA	Dual-Energy X-ray Absorptiometry
FEV1	Forced Expiratory Volume in 1 Second
FVC	Forced Vital Capacity
GOLD	Global Initiative for Chronic Obstructive Lung Disease
HH	Hiatal Hernia
HRCT	High-Resolution Computed Tomography
HU	Hounsfield Units
IBM	International Business Machines
ILA	Interstitial Lung Abnormalities
ILD	Interstitial Lung Disease
kVp	Kilovolt Peak
mAs	Milliampere-seconds
mMRC	modified Medical Research Council (dyspnea scale)
PA/Ao	Pulmonary Artery to Ascending Aorta Diameter Ratio
PAD	Pulmonary Artery Diameter
PAE	Pulmonary Artery Enlargement
PASP	Pulmonary Artery Systolic Pressure
PH	Pulmonary Hypertension
RHC	Right Heart Catheterization
SPSS	Statistical Package for the Social Sciences
VB	Virtual Bronchoscopy
WHO	World Health Organization
